# Strategies to Reduce Biofilm Formation in PEEK Materials Applied to Implant Dentistry—A Comprehensive Review

**DOI:** 10.3390/antibiotics9090609

**Published:** 2020-09-16

**Authors:** Renata Scheeren Brum, Luiza Gomes Labes, Cláudia Ângela Maziero Volpato, César Augusto Magalhães Benfatti, Andrea de Lima Pimenta

**Affiliations:** 1 Center for Research on Dental Implants (CEPID), Dentistry Department (ODT), Federal University of Santa Catarina (UFSC), Rua Delfino Conti, 1240, Campus Universitário—Trindade, Florianópolis SC 88036-020, Brazil; luiza_labes@hotmail.com (L.G.L.); claudia.m.volpato@ufsc.br (C.Â.M.V.); 2 Integrated Laboratories Technologies (InteLab), Department Chemical Engineering (EQA), Federal University of Santa Catarina (UFSC), Florianópolis SC 88040-970, Brazil; andrea@intelab.ufsc.br; 3 Department of Biology, ERRMECe, Université de CergyPontoise, MaisonInternationale de la Recherche, Rue Descartes, CEDEX, 95000 Neuville sur Oise, France

**Keywords:** biofilms, biofilm inhibition, dental implants, bacteria, peri-implantitis, polyether-ether-ketone

## Abstract

Polyether-ether-ketone (PEEK) has emerged in Implant Dentistry with a series of short-time applications and as a promising material to substitute definitive dental implants. Several strategies have been investigated to diminish biofilm formation on the PEEK surface aiming to decrease the possibility of related infections. Therefore, a comprehensive review was carried out in order to compare PEEK with materials widely used nowadays in Implant Dentistry, such as titanium and zirconia, placing emphasis on studies investigating its ability to grant or prevent biofilm formation. Most studies failed to reveal significant antimicrobial activity in pure PEEK, while several studies described new strategies to reduce biofilm formation and bacterial colonization on this material. Those include the PEEK sulfonation process, incorporation of therapeutic and bioactive agents in PEEK matrix or on PEEK surface, PEEK coatings and incorporation of reinforcement agents, in order to produce nanocomposites or blends. The two most analyzed surface properties were contact angle and roughness, while the most studied bacteria were *Escherichia coli* and *Staphylococcus aureus*. Despite PEEK’s susceptibility to biofilm formation, a great number of strategies discussed in this study were able to improve its antibiofilm and antimicrobial properties.

## 1. Introduction

The diverse microbiome that harbors in the oral cavity plays an important role in health maintenance through the development of the immune response and inhibition of the pathogen colonization [1]. However, under certain circumstances, normal microbiota may be responsible for many oral diseases [2,3]. Oral dysbiosis triggers important changes, reducing the number of beneficial bacteria and favoring the growth of potential pathogens [4]. This is particularly worrying in susceptible individuals affected by periodontitis, a biofilm related disease characterized by alveolar bone resorption, which may lead to tooth mobility and tooth loss [5,6]. In fact, periodontal patients who were rehabilitated with dental implants are more predisposed to develop peri-implant diseases, for which poor plaque control also acts as a primary etiologic factor [7].

In a systematic review carried out in 2017, [8] patient-level data and implant-level data indicated that peri-implantitis was present in 9.25% and 19.83% of analyzed cases, respectively, while mucositis affected 29.48% of patients and 46.83% of implants analyzed [8]. Since there was no consensus on the best treatment protocol [9], biofilm prevention becomes not only desirable but necessary [10]. This can be achieved at the clinical level through favorable implant position and adequate prosthetic design, accompanied by oral hygiene education and regular appointments [10]. Still, at the research level, there is an incessant demand for investigations to develop materials with either antibiofilm or antimicrobial surfaces, or both, through manipulation of surface topographical properties (i.e., contact angle and roughness), or by the incorporation of antibiofilm agents, which can be evaluated through physicochemical analysis [11,12,13,14].

Since the demonstration of titanium osseointegration by Branemark et al. (1981) [15], this material has been widely used in Implant Dentistry, revolutionizing oral rehabilitation modalities [16]. However, under certain circumstances, such as therapeutic treatment of peri-implantitis [17] or wear-corrosion, metallic debris is released resulting in prejudicial effects to peri-implant tissues. It had been proved that those metal particles stimulate molecular mechanisms such as enhancement of proinflammatory cytokines and osteoclasts activity, as well as infiltration of inflammatory cells with cytotoxic and genotoxic effects [18]. Hence, there is a growing interest in the development of an alternative material that can be used in dental implants and as implant abutments [19,20,21]. 

Within this context, the thermoplastic biocompatible polymer polyether-ether-ketone (PEEK) stands out, with several desired properties to Implant Dentistry improvements, such as mechanical and chemical resistance, stability at high temperatures (enabling sterilization) and natural white pigmentation (favorable for esthetics) [22,23]. Several methods have been studied to establish an effective adhesion of PEEK to resin-matrix composite in restorative dentistry, which is useful to the esthetic of provisional restorations [24]. Moreover, its production process is very versatile, as PEEK is compatible with many reinforcement agents and surface coatings, which can be used to improve its mechanical and biological properties [25,26,27]. Currently, PEEK is safely used in Implant Dentistry as provisional abutments, healing screws, prosthetic transfers and frameworks [20,23,28]. Nevertheless, as reported by Khonsari et al. [29], there are cases in which PEEK dental implants had been employed in patients and poor osseointegration led to severe infectious complications and subsequent implant loss.

**Figure 1:**
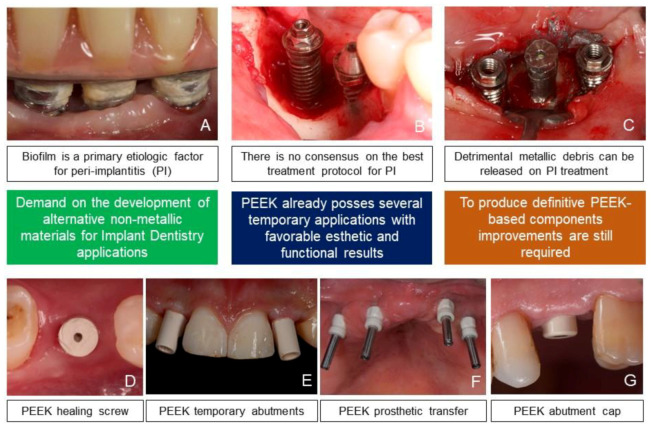
(**A**) Biofilm formation on titanium implants, underneath an implant-supported total prosthesis; (**B**) bone defects around dental implants at posterior lower jaw, a sequel of peri-implantitis; (**C**) metallic debris being released to peri-implant tissues during peri-implantitis treatment (implantoplasty); (**D**) PEEK healing screw (FGM, Brazil); (**E**) PEEK temporary abutments (Straumann, Switzerland) that support esthetic restorations; (**F**) PEEK prosthetic transfers (FGM, Brazil); (**G**) PEEK abutment cap (Straumann, Switzerland).

Figure 1 ilustrates the propositions exposed above. illustrates the propositions exposed above. In order to develop a PEEK-based dental implant or even to convert the available applications from provisional to definitive (i.e., PEEK-based prosthetic components), additional research is necessary. Therefore, a comprehensive literature review was carried out aiming to investigate available strategies to reduce biofilm formation on PEEK materials for Implant Dentistry applications. 

## 2. Strategies to Reduce Biofilm Formation in PEEK Materials Applied to Implant Dentistry

A full strategy with inclusion and exclusion criteria, as well as the flow chart of selected studies, are available as Supplementary Data. From a total of 376 studies initially found during the literature search, 33 were chosen for full text reading based on titles. Thereafter, 31 studies fulfilled the inclusion criteria of this review. Table 1 and Table 2 reveal comprehensive information on pure and modified PEEK, respectively.

### 2.1. Study Characteristics

Amongst the included studies, 5 involved in vitro associated to in vivo (animal) investigations, while 26 were restricted to in vitro studies. In vivo (human) studies did not fulfill inclusion criteria of this review. Regarding PEEK modification strategies, 6 studies analyzed pure PEEK compared to other materials [30,31,32,33,34,35] (e.g., titanium, silicon, gold, silver, zinc oxide, zirconia, silicon nitride) and none of them revealed special antibiofilm or antimicrobial properties of PEEK material. A total of 25 studies used strategies to reduce biofilm and bacterial colonization on PEEK, which were able to successfully confer either antibiofilm or antimicrobial properties, or both, to the material. Regarding applications aimed at the investigated materials, orthopedic, dental and the treatment of bone defects were the most commonly mentioned, followed by the development of biomaterials in general.

### 2.2. Available Strategies to Reduce Biofilm Formation on PEEK Materials

**Figure 2:**
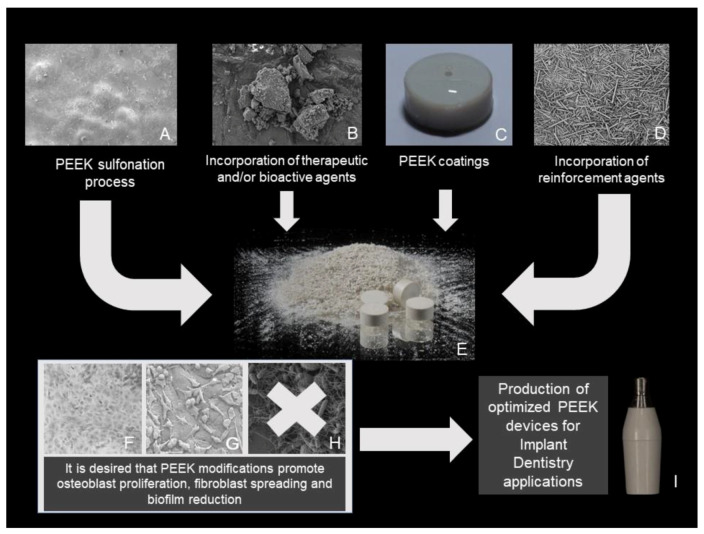
Summary of some available strategies to improve PEEK biological properties. (**A**) SEM image of a sulfonated PEEK membrane; (**B**) SEM image of bioglass particles; (**C**) photography of PEEK coated with adhesive film; (**D**) SEM image of natural amorphous silica fibers; (**E**) photography of PEEK powder and PEEK cylinders manufactured through compression molding; (**F**,**G**) SEM images of MC3T3 osteoblasts on zirconia surface; L929 fibroblasts on PEEK surface; (**H**) undesired biofilm formation on material surface; (**I**) PEEK provisional abutment (Straumann, Switzerland).

Strategies are summarized and illustrated at Figure 2 and are listed as follows:

(a) PEEK sulfonation process, which can be employed either to produce a 3D network on polymer surface [39], or to embed therapeutic compounds (e.g., lactams [45,46], mouse beta-defensin [59]). Further surface treatments were also employed after the sulfonation process, such as chlorogenic acid/grafting peptide [43], graphene oxide coating [61] and hydrothermal treatment [48].
(b) Incorporation of therapeutic and/or bioactive agents in the PEEK matrix or on the PEEK surface, such as simvastatin-PLLA [39]; Ag and Zn ions [37,39,40,58], dexamethasone plus minocycline-loaded liposomes [57], bioactive titanium dioxide (TiO_2_) [52], 2-methacryloyloxyethyl phosphorylcholine [51] and titanium plasma [56].
(c) PEEK coatings, such as the hybrid coating of titanium dioxide and polydimethylsiloxane [52]; chitosan/bioactive glass/lawsone [53], red and gray selenium nanoparticles [56], mussel-inspired polydopamine with silver nanoparticles incorporated and silk fibroin gentamicin sulfate [57,58].
(d) Incorporation of reinforcement agents to produce nanocomposites and/or blends (carbonylated PEEK grafted to ZnO45, PEEK/poly-ether-imide blends [41], carbon fiber reinforced PEEK further treated with oxygen plasma [44], PEEK/nano-fluorohydroxyapatite [54], nano-bioglass/PEEK [60]).

### 2.3. Microbiological Analysis

The most commonly investigated bacteria were *Escherichia coli* and *Staphylococcus aureus*, but other microorganisms such as *Streptococcus sanguinis*, *Streptococcus oralis*, *Streptococcus faecalis*, *Streptococcus gordonni*, *Streptococcus epidermidis*, *Pseudomonas aeruginosa*, *Aggregatibacter actinomycetemcomitans*, *Porphyromonas gingivalis*, *Fusobacterium nucleatum*, *Enterococcus faecalis*, *Candida albicans*, *Actinomyces naeslundii*, *Streptococcus mutans* and *Staphylococcus epidermidis* were also studied. Microbiological analysis was very heterogenic, and several methods were used, which are summarized in Table 1 and Table 2. Among the included methods, it should be highlighted that the most recurrent ones were plate-counting, for the determination of average colony forming units (CFU/mm^2^); Real-Time Polymerase Chain Reaction (RT-PCR) and Live/Dead cells analysis, followed by FE-SEM and confocal laser scanning microscopy; bacterial growth inhibition zone tests; crystal violet assays; longevity and stability of antibacterial activity and agar diffusion assay.

### 2.4. Physicochemical and Topographical Characterization

With the exception of 7 papers [32,37,40,41,45,56,60], all the other studies analyzed surface topographical aspects, such as either or both contact angle and surface roughness. The physicochemical and additional characterization of included papers was achieved by energy-dispersive X-ray spectroscopy (EDX), X-ray photoelectron spectroscopy (XPS), porosity evaluation through drainage method, dynamic differential scanning calorimetry (DSC), X-ray diffractograms (XRD), Hydrogen nuclear magnetic resonance (^1^H-NMR), thermogravimetric analysis (TGA), Fourier-transform infrared spectroscopy (FTIR) and UV spectrophotometer.

## 3. Discussion

Investigations have demonstrated that peri-implantitis is a heterogeneous infection, in which periodontopathogens and opportunistic microorganisms act simultaneously [62,63,64]. Moreover, the disease has been associated to specific immunological alterations on peri-implant crevicular fluid levels of proinflammatory, anti-inflammatory and osteoclastogenesis-related chemokines [65]. Several studies analyzed in this review [30,31,32,33,34,35] investigated biofilm and antimicrobial properties of pure PEEK, demonstrating that the polymer is susceptible to biofilm colonization. Within a context in which PEEK clinical applications in Implant Dentistry are increasing [28], strategies to modify its surface to enhance its antimicrobial/antibiofilm properties are crucial.

It becomes even more important to improve PEEK materials with the above-mentioned properties when considering that biofilms are organized polymicrobial communities that offer bacteria protection against environmental factors and antibiotic treatments [66,67]. In vitro analysis of submucosal biofilm samples of 120 peri-implantitis sites revealed that 71.7% exhibited bacterial pathogens resistance to one or more of tested antibiotics (clindamycin, amoxicillin, doxycycline or metronidazole) [68]. Therefore, the identification of compounds capable of inhibiting biofilm formation or disrupt biofilm organization emerges as an attractive alternative to avoid peri-implant related infections [69,70]. It is important to notice that this approach is not expected to completely eliminate biofilm formation, but it is a very effective way of modifying oral ecology instead, reducing the number of pathogenic bacteria and favoring the growth of mutualistic species. By doing so, the host organism is provided with just the necessary advantage to defeat the pathogens using its own resources.

Additionally, it is important to analyze PEEK surface properties and its influence on biologic systems. For example, the PEEK hydrophobic surface associated to its bio inertness is a major concern when prospecting for the expansion of its application in Implant Dentistry [28,71], as this type of surface typically reduces cellular adhesion and does not promote osseointegration [22]. Numerous modifications have been proposed to overcome those limitations, such as blending with bioactive particles such as titanium dioxide, hydroxyapatite and fluorapatite [72,73,74]. Interestingly, the present review exposed that some of those strategies showed the favorable additional effect of reducing biofilm formation [41,44,54]. For example, a very promising candidate to replace metallic implants is carbon fiber reinforced PEEK (CFRPEEK) [75], which has similar elastic modulus to the human cortical bone [22]. One of the studies included in this review [44] proposed a dual zinc and oxygen plasma immersion ion implantation to modify CFRPEEK. Despite the fact that this strategy made the surface far more hydrophobic (contact angle shifted from 66.6° to 144.1° after surface modification), it also improved both osteogenic and antibacterial activities, as evaluated through MC3T3-E1 and rat bone mesenchymal stem cell development and through *Staphylococcus aureus*, *MRSA* and *Staphylococcus epidermidis* inhibition [44]. Those findings provide positive perspectives of the development of PEEK surfaces enhanced with bioactive and antibiofilm properties, which is favorable for PEEK-based dental implant development.

Bone cell activity on the PEEK surface is very important to achieve proper osseointegration on dental implants, but considering that an imperative application for PEEK in Implant Dentistry is as implant abutments [76], the gingival sealing must be analyzed as well, since it provides protection to implants against infections by potential pathogens [10]. Among the studies included in this review describing strategies for PEEK modification through the incorporation of antibiofilm agents, the embedding of lactams through the PEEK sulfonation process is worth mentioning [45]. Lactams are compounds analogous to furanones, which were initially isolated from the algae *Delisea pulchra*, and had been proved to be effective against *Streptococus mutans* biofilms [77]. An in vitro study [26] demonstrated that PEEK sulfonation positively interferes with the ability of fibroblasts L929 to spread over the surface of the material [26]. This corroborates previous indications that PEEK sulfonation is a suitable process for the development of modified PEEK abutments with embedded antibiofilm compounds.

In addition to the mentioned in vitro studies indicating these strategies as promising approaches to develop clinical materials biofilm resistant, an in vivo (human) investigation also revealed that PEEK healing abutments did not affect important parameters of peri-implant health, such as marginal bone loss and soft tissue recession, during a three-month evaluation period [78]. Therefore, it seems plausible to associate PEEK inherent favorable properties with adequate strategies to maximize its biological properties and consequently achieve even better clinical outcomes in the near future.

## 4. Conclusions

Within the scope of the present review, it may be concluded that pure PEEK is susceptible to biofilm formation and that several strategies presented here are able to significantly improve its antibiofilm and antimicrobial properties. Those strategies include the PEEK sulfonation process, incorporation of therapeutic and/or bioactive agents in the PEEK matrix or on the PEEK surface, PEEK coatings and incorporation of reinforcement agents to produce nanocomposites and/or blends. Since the use of PEEK in Implant Dentistry is increasing, those modifications are necessary in order to enable patients to benefit from these new materials which present great potential to prevent infections. Therefore, it is expected that further in vivo studies, both in animals and humans, will make available PEEK-based dental implants and improved implant abutments for clinical practice applications.

## Figures and Tables

**Table 1:** Descriptive analysis of unmodified PEEK materials ^a^.

Reference	Materials (Roughness and Contact Angle Values) ^b^	Microorganisms	Microbiologic Assay	Biologic Response
1. Barton et al. [30]	✓ Poly(orthoesther) (78°); ✓ Poly(L-lactic acid) (84°); ✓ PEEK (90°); ✓ Polysulfone (84°); ✓ High molecular weight polyethylene (106°);	✓ *Staphylococcus epidermidis*; ✓ *Pseudomonas aeruginosa*; ✓ *Escherichia coli*;	✓ Bacteria adhesion with or without hyaluronic acid;	Bacterial adhesion was higher on PEEK than on biodegradable polymers;
2. Bock et al. [31]	✓ PEEK (1.034 nm; 86°); ✓ Si_3_N_4_ (1094 nm; 28°); ✓ Af-Si_3_N_4_ (830 nm; 66°); ✓ Ox-Si_3_N_4_ (745 nm; 8°); ✓ N_2_-Si_3_N_4_ (654 nm; 9°); ✓ Ti_6_Al_4_V (494 nm; 71°);	✓ *Escherichia coli*; ✓ *Staphylococcus epidermidis*;	✓ Bacterial detachment and counting: average colony forming units (CFU/mm^2^)	For both bacteria and at both experimental times biofilm growth was greater on PEEK;
3. Bressan et al. 2017 [32]	✓ Taper cap gold coping; ✓ PEEK coping ✓ Copings were connected to dental implants;	✓ *Aggregatibacter actinomycetemcomitans*; ✓ *Porphyromonas gingivalis*; ✓ *Fusobacterium nucleatum*;	✓ Real-time polymerase chain reaction (PCR); ✓ Visual assessment;	No significant differences between groups were identified;
4. Gorth et al. [33]	✓ PEEK (1 nm); ✓ Titanium (3 nm); ✓ Si_3_N_4_ (25 nm) ✓ Si_3_N_4_ polished (10 nm);	✓ *Staphylococcus epidermidis*; ✓ *Pseudomonas aeruginosa*; ✓ *Staphylococcus aureus*; ✓ *Escherichia coli*; ✓ *Enterococcus*;	✓ Bacterial function: crystal violet staining and a Live/Dead assay;	Exponential growth of biofilm was noted on PEEK when exposed to *S. epidermidis, S. aureus, P. aeruginosa and E. coli*. With the exception of *Enterococcus*, biofilm formation was lower on titanium compared to PEEK for time periods >48 h. PEEK showed the highest biofilm affinity;
5. Hahnel et al. 2015 [34]	✓ Zirconia (0.16 μm); ✓ Titanium (0.17 μm); ✓ PEEK (0.04 μm); ✓ Polymethylmethacrylate—PMMA (0.05 μm);	✓ *Candida albicans*; ✓ *Streptococcus mutans*; ✓ *Actinomyces naeslundii* ✓ *Streptococus gordonii*;	✓ Biofilm analysis: MTT-based cell viability assay and Live/Dead BacLight bacterial viability kit solution. ✓ Analysis on fluorescence microscope;	The lowest quantity of adherent viable biomass was identified on the surface of PEEK compared to other groups. After 44 h, biofilms on zirconia yielded the highest value of dead microorganisms and PMMA yielded the lowest value;
6. Webster et al. [35]	✓ Si_3_N_4_ (39°); ✓ ASTM grade 4 titanium (76°); ✓ PEEK (95°);	✓ *Staphylococcus epidermidis*;	✓ Bacterial infection and bone growth: histologic quantification for the number of bacteria in the implant area and juxtaposed to the implant;	Live bacteria were identified around PEEK (88%) and Ti (21%) implants, while none were observed adjacent to Si_3_N_4_;

^a^ A decrease in free energy favors stability. ^b^ Contact angle ≥ 90° means that the material is hydrophobic, and <90°means that it is hydrophilic.

**Table 2:** Descriptive analysis of modified PEEK materials.

Reference	PEEK Modification Strategy	Materials (Roughness and Contact Angle Values)	Microorganisms	Microbiologic Assay	Biologic Response
1. Barkarmo et al. [36]	PEEK blasting;	✓ PEEK (0.57 μm; 70.33°); ✓ Blasted PEEK (1.85 μm; 108.36°); ✓ Titanium Grade 4 (0.23 μm; 62.43°); ✓ Ti_6_Al_4_V (0.28 μm; 58.82°);	✓ *Streptococcus sanguinis*; ✓ *Streptococcus oralis*; ✓ *Enterococcus faecalis*; ✓ *Streptococcus gordonii*;	✓ Modification of the original method of Christensen et al. (1985).	Bacteria showed increased biofilm formation on blasted PEEK (exception: *E. faecalis*—higher on cp-Ti compared with other materials).
2. Deng et al. [37]	Novel Ag-decorated 3D printed PEEK via catecholamine chemistry;	✓ PEEK scaffolds fabricated layer by layer; ✓ PEEK coated with a pDAnanolayer by dopamine solution and immersion in AgNO_3_ with subsequent UV light treatment;	✓ *Escherichia coli*; ✓ *Staphylococcus aureus*;	✓ Evaluation of bacterial dynamics curves; ✓ Antibiofilm formation;	PEEK scaffold pDA-coated and UV-treated had significant contact and release killing capacities. Biofilms were reduced in the presence of silver;
3. Deng et al. [38]	Hierarchically micro/nanoscale produced on PEEK and a simvastatin-PLLA film-tobramycin microspheres delivery system was fabricated;	✓ PEEK; ✓ NSPEEK (treatment with mixed acid H_2_SO4:HNO3); ✓ NSP/SIM(1 mm)-PLLA (additional immersion in SIM solution) (93°); ✓ NSP/SIM (1 mm)-TOB: additional emulsion 3% of PLLA in CH_2_Cl_2_; 0.3% of TOB in ultra-pure water) dropped and spin-coated (84°);	✓ *Escherichia coli*; ✓ *Staphylococcus. aureus*;	✓ Agar diffusiontest; ✓ Bacteria Adhesion; ✓ Antibiofilm Tests; ✓ Evaluation through Live/Dead kits, FE-SEM and confocal laser scanning microscopy;	Few bacteria were detected on the NSP/SIM (1 mm)-TOB group, while other groups had plenty of bacteria adhered. PEEK and NSPEEK showed uncontrolled biofilm proliferation, while no biofilm was observed on NSP/SIM (1 mm)-TOB group;
4. Deng et al. [39]	Dual therapy implant coating developed on the 3D micro-/nanoporous sulfonated PEEK via layer-by-layer self-assembly of Ag ions and Zn ions;	✓ SPEEK: sulfonated PEEK (83.75°); ✓ Ag-SPEEK: SPEEK further treated by chitosan solution, and by Ag ion-sodium alginate solution; ✓ Zn-SPEEK: assembling of Zn ion–containing chitosan with pure sodium alginate; ✓ Ag/Zn-SPEEK: Ag-SPEEK further exposed to UV/ozone;	✓ *Escherichia coli*; ✓ *Staphylococcus aureus*;	✓ Antibacterial Kinetic Tests; ✓ Determination of CFU; ✓ Bacterial Growth Inhibition Zone Tests; ✓ SEM Characterization of Bacteria;	Ag–SPEEK substrate was superior regarding antibacterial properties against *E. coli,* while absence of obvious antibacterial effects against *S. aureus* was observed;
5. Díez-Pascual et al. [40]	Production of nanocomposites via melt-blending, by addition of a carboxylated polymer derivative covalently grafted onto the surface of hydroxyl-terminated ZnO nanoparticles;	✓ PEEK, PEEK/ZnO (nanoparticle content: 1.0); ✓ PEEK/ZnO (2.5); ✓ PEEK/ZnO (5.0); ✓ PEEK/COOH; ✓ PCOZnO, PEEK/ PCOZnO (1.0); ✓ PEEK/ PCOZnO (2.5); ✓ PEEK/ PCOZnO (5.0); ✓ Obs:PCOZnO is PEEK−CO−O−CH2−ZnO;	✓ *Escherichia coli*; ✓ *Staphylococcus. aureus*;	✓ CFU/sample calculation;	Nanocomposites with polymer-grafted nanoparticles exhibited superior antibacterial activity against both studied bacteria. This effect increased upon raising nanoparticle content and was stronger on *E. coli*;
6. Díez-Pascual et al. [41]	Production of biocompatible ternary nanocomposites based on poly PEEK/poly(ether-imide) (PEI) blends reinforced with bioactive titanium dioxide (TiO_2_) nanoparticles via ultrasonication followed by melt-blending;	✓ TiO_2_; ✓ PEEK; ✓ PEI; ✓ PEEK/PEI; ✓ PEEK/PEI/ TiO_2_ (1.0 wt %); ✓ PEEK/PEI/ TiO_2_ (4.0 wt %); ✓ PEEK/PEI/ TiO_2_ (8.0 wt %) UV irradiated;	✓ *Escherichia coli*; ✓ *Staphylococcus aureus*;	✓ Survival ratio calculation under presence or absence of UV light against bacteria;	The nanoparticles conferred antibacterial action versus tested bacteria in the presence and in the absence of UV light. The highest inhibition was attained at 4.0 wt % nanoparticle concentration;
7. Gan et al. [42]	Nitrogen plasma immersion ion implantation (PIII) on PEEK;	✓ PEEK-C (50.6 nm; 84.5°), ✓ PEEK-I: N_2_, no voltage, no pulse width and no frequency—90 min (435.9 nm; 19.93°), ✓ PEEK-L: N_2_, −20 kV of voltage, pulse width of 30 uS, frequency of 1000 W—90 min (443.23 nm; 20.67°), ✓ PEEK-H: N_2_, −20 kV of voltage, pulse width of 50 uS, frequency of 1000W—90 min (608.4 nm; 17.74°);	✓ *Staphylococcus aureus*;	✓ Colony-counting and plate-counting methods;	The number of colonies adherents on the PEEK-L and PEEK-H was lower than that on PEEK-C and PEEK-I. Nitrogen PIII using high pulse or low pulse inhibited *S. aureus* early adhesion on PEEK, which exhibited antibacterial property;
8. He et al. [43]	Drug-loaded (chlorogenic acid, CGA)/grafted peptide (BFP) hydrogel system supported on a sulfonated PEEK (SPEEK) surface, using sodium alginate (SA);	✓ SPEEK (67.75°), ✓ SPEEK@SA (23.33°) ✓ SPEEK@SA-CGA—(30.5°), ✓ SPEEK@SA(CGA)BFP (28.08°);	✓ *Escherichia coli*; ✓ *Staphylococcus aureus*;	✓ Evaluation through plate-counting method after inoculation and incubation.	SPEEK and SPEEK@SA did not inhibit *E. coli* growth. SPEEK@SA(CGA) and SPEEK@SA(CGA)BFP scaffolds had a noticeable antibacterial effect on both tested bacteria;
9. Lu et al. [44]	Dual zinc and oxygen plasma immersion ion implantation (Zn/O-PIII) applied to modify carbon fiber reinforced PEEK (CFRPEEK);	✓ CFRPEEK (66.6°); ✓ CFRPEEK + oxygen plasma immersion ion implantation (Zn/O-PIII) (144.1°);	✓ *Escherichia coli*; ✓ *Staphylococcus. aureus*; ✓ *Pseudomonas aeruginosa*; ✓ Methicillin-resistant *Staphylococcus aureus*; ✓ *Staphylococcus. epidermidis*; ✓ Biofilm-negative *Staphylococcus epidermidis*;	✓ Antibacterial activity: bacterial counting method; ✓ Morphology of the adhered bacteria: SEM;	*S. aureus*, MRSA and *S. epidermidis* reduction on Zn/O-PIII-CFRPEEK is over 95% at 24 h. This group showed no antibacterial effect on *S. epidermidis* (biofilm-negative strain), *E. coli* and *P. aeruginosa;*
10. Montero et al. [45]	PEEK sulfonation treatment to functionalize and embed therapeutical substances (lactam);	✓ Sulphonated-PEEK without lactams embedded, ✓ Sulphonated-PEEK with lactams embedded;	✓ *Streptococcus mutans*;	✓ Evaluation through plate-counting method after inoculation and incubation (biofilm and planktonic); ✓ Bacterial morphology: SEM;	Planktonic growth showed no significant difference between groups, while biofilm inhibition was found comparing SPEEK with lactams. *S. mutans* biofilm grew widely separately as agglomerates on SPEEK without lactams, while it could not be detected on SPEEK with lactams;
11. Montero et al. [46]	PEEK sulfonation (SPEEK) on various degrees (SD);62%, G2 68%, G3 90%, G4 75% and G5 69%	✓ SPEEK (50 °C, 1 h, SD: 62%); ✓ SPEEK (50 °C, 1.5 h, SD: 68%); ✓ SPEEK (50 °C, 2 h, SD: 90%); ✓ SPEEK (50 °C, 2.5 h, 75%); ✓ SPEEK (50 °C. 3 h, SD: 69%);	✓ *Streptococcus mutans*; ✓ *Enterococcus faecalis*;	✓ Evaluation through plate-counting method after inoculation and incubation (biofilm and planktonic);	SPEEK heated for 3 h was the group with lowest values of planktonic growth;CFU from *S. mutans* biofilm showed a significant decrease on SPEEK sulfonated for 2, 2.5 and 3 h. *E. faecalis* showed this reduction only on groups sulfonated for 2.5 and 3 h;
12. Ouyang et al. [47]	Preparation of graphene oxide (GO) modified SPEEK (GO-SPEEK) through dip-coating method;	✓ PEEK (91.2°); ✓ SPEEK (103.9°); ✓ 0.5 GO-SPEEK (57°); ✓ 1 GO-SPEEK (47.7°);	✓ *Escherichia coli*; ✓ *Staphylococcus aureus*;	✓ Live/Dead fluorescence imaging (Confocal laser scanning microscope-CLSM evaluation);	0.5 GO-SPEEK and 1 GO-SPEEK groups exhibit proper antibacterial properties against *E. coli*, but poor against *S. aureus*;
13. Ouyang et al. [48]	PEEK was sulfonated by concentrated sulfuric acid to fabricate a three-dimensional (3D) network with hydrothermal treatment subsequently;	✓ PEEK (86°); ✓ SPEEK (110°); ✓ SPW25 (110°); ✓ SPW120 (110°);	✓ *Escherichia coli*; ✓ *Staphylococcus aureus*;	✓ Incubation according the standard of Luria–Bertani; Bacteria morphology: SEM;	Amounts of *E.coli* were reduced to o 100%, 100%, and 24% on SPEEK, SPW25 and SPW120, respectively. On the same groups *S. aureus* was reduced by nearly 100%.
14. Rochford et al. [49]	Injection moulded (PO) or machined (PA) PEEK exposed to an oxygen gas plasma in a plasma cleaner;	✓ Injection molded PEEK (PO) (85 nm, 83°); ✓ Injection molded PEEK machined (PA) (536 nm, 73°); ✓ Commercially purê micro-rough titanium (Ti) (530 nm, 68°); ✓ Treated side of sterile Thermanox Txh (7.5 nm, 67°);	✓ *Staphylococcus aureus*; ✓ JAR (bothclinicalisolates) ✓ *Staphylococcus epidermidis*;	✓ Bacterial adhesion quantification: adhesion chamber biofilm reactor;	Surface modification of PEEK did not lead to a significant change in bacterial adhesion in the preoperative contamination model. In the postoperative contamination model, *S. aureus* adhesion was increased on the modified surfaces. *S. epidermidis* adhesion to modified PEEK was lower than to nonmodified PEEK in the postoperative model;
15. Rochford et al. [50]	PEEK films were oxygen plasma treated to increase surface free energy;	✓ PEEK (28 nm, 81°); ✓ PEEK exposed to oxygen gas plasma for 900 s (21 nm, 53°); ✓ PEEK exposed to oxygen gas plasma for 1800 s (15 nm, 51°);	✓ *Staphylococcus epidermidis*; ✓ *Staphylococcus aureus*;	✓ Bacterial adhesion was assessed using a parallel plate flow chamber and camera;	There was no significant difference in bacterial adhesion between treated and untreated surfaces;
16. Tateishi et al. [51]	Modified PEEK surface by photoinduced and self-initiated graft polymerization with 2methacryloyloxyethyl phosphorylcholine, under radiation UV;	✓ Untreated PEEK; ✓ PMPC-grafted PEEK;	✓ *Escherichia coli*;	✓ Number of bacteria adhered on the surface was countered from the SEM images;	SEM revealed adhered bacteria on PEEK, whereas no bacterium was observed on the PMPC-grafted PEEK;
17. Tran et al. [52]	Production of a hybrid coating of titanium dioxide and polydimethylsiloxane (PDMS) to regulate silver releasing;	✓ PEEK (90°), ✓ Coated PEEK in H50 volume and 38.4 μL Ag (H50-38.4) (>120°); ✓ Coated PEEK in H50 volume and 384 μL Ag (H50-384) (>120°); ✓ Coated PEEK in H75 volume and 38.4 μL Ag (H75-38.4) (>120°); ✓ Coated PEEK in H75 volume and 384 μL Ag (H75-384) (>120°); ✓ Coated PEEK in H95 volume and 38.4 μL Ag (H95-38.4) (>120°); ✓ Coated PEEK in H95 volume and 384 μL Ag (H95-384) (>120°);	✓ *Staphylococcus aureus*; ✓ *Staphylococcus epidermidis*;	✓ Antibacterial property: Kirby–Bauer tests; ✓ Biofilm growth: after incubation samples were analyzed by SEM;	Higher Ag loadings resulted in a significant increase in the diameter of the bacteria inhibition zone. On PEEK, a thick and dense biofilm was formed. On H50-38.4, H75-38.4 and H95-38.4 smaller colonies of *S. aureus* were found, while in H50-384, H75-384 and H95-384 no bacterial colonies were found;
18. Ur Rehman et al. [53]	Chitosan/bioactive glass (BG)/lawsone coatings were deposited by electrophoretic deposition (EPD) on polyetheretherketone (PEEK)/BG layers (previously deposited by EPD on 316-L stainless steel);	✓ PEEK/BG (2.2 μm, 100°), ✓ Chitosan/BG/lawsone (1.3 μm, 45°), ✓ Stainless steel chitosan/BG/lawsone and PEEK/BG coated (multilayered);	✓ *Staphylococcus carnosus*;	✓ Inhibition zones were measured using ‘ImageJ’ analysis;	Chitosan/BG/lawsone and the stainless steel chitosan/BG/lawsone PEEK/BG coated induced inhibition halo against *S. carnosus*. Halo zone was wider for the multilayered group (10 mm vs 4 mm);
19. Wang et al. [54]	Development of a PEEK/nano-fluorohydroxyapatite (PEEK/nano-FHA) biocomposite;	✓ PEEK (83.5°); ✓ PEEK/nano-fluorohydroxyapatite (PEEK/nano-FHA) (71.5°);	✓ *Streptococcus mutans*	✓ Microbial ViabilityAssay Kit; ✓ Biofilm formation assay: LIVE/DEAD BacLight bacterial viability kit and evaluation on CLSM;	PEEK/nano-FHA biocomposite inhibited bacterial adhesion and proliferation, which did not occur with PEEK;
20. Wang et al. [55]	PEEK coated with red and gray selenium nanoparticles through a quick precipitation method;	✓ Red selenium nanoparticles as coatings for PEEK (78.148°); ✓ Grey selenium nanoparticles as coatings for PEEK (76.988°); ✓ PEEK without selenium coatings (68.478°);	✓ *Pseudomonas aeruginosa*;	✓ Bacterial inhibition: crystal violet assays;	Red and gray selenium-coated PEEK significantly inhibited the growth of *P. aeruginosa* compared with uncoated PEEK at all experimental times.
21. Wang et al. [56]	Titanium plasma immersion ion implantation (PIII) technique was applied to modify the carbon-fiber-reinforced polyetheretherketone (CFRPEEK) surface, constructing a unique multilevel TiO_2_ nanostructure;	✓ CFRPEEK; ✓ CFRPEEK modified with titanium plasma immersion ion implantation (PIII) technique (Ti-120);	✓ *Streptococcus mutans*; ✓ *Fusobacterieun nucleatum*; ✓ *Porphyromonas gingivalis*;	✓ Live/Dead BacLight bacteria viability kits and evaluation at confocal laser-scanning microscope; ✓ Morphologicalobservation: SEM; ✓ Longevity and stability of antibacterial activity;	The TiPIII modified surface can reduced *S. mutans, F. nucleatum* and *P. gingivalis* adhesion and growth, directly implicating on death of adhesive bacterial;
22. Xu et al. [57]	PEEK modified surface using dexamethasone plus minocycline-loaded liposomes (Dex/Mino liposomes) bonded by a mussel-inspired polydopamine coating (pDA);	✓ PEEK (22.25 nm, 71°); ✓ PEEK-pDA (53.33 nm, 24°); ✓ PEEK blanklipossomes (35.90 nm, 61°);	✓ In vitro: *Streptococcus mutans* and *Porphyromonas gingivalis*;	✓ The Microbial Viability Assay Kit-WST and LIVE/DEAD BacLight Bacterial Viability Kit (CLSM evaluation); ✓ Cell morphology imaging;	Minor releasing from PEEK blank lipossomes surfaces effectively prevented bacterial adhesion and proliferation. The antibacterial efficiency of PEEK blank lipossomes was about 97.4% against *S. mutans*;
23. Yan et al. [58]	A mussel inspired self-polymerized polydopamine (PDA) with silver nanoparticles (AgNPs) incorporated and silk fibroin (SF)/ gentamicin sulfate (GS) coating was constructed upon porous PEEK surface;	✓ PEEK (65°); ✓ SPEEK (81°); ✓ SP-PDA (49°); ✓ SP-PDA-Ag (without UV); ✓ SP-PDA-Ag (46°); ✓ SP-PDA-Ag/GS-Silk (56°);	✓ *Staphylococcus aureus*; ✓ *Escherichia coli*;	✓ Antibacterial assay: Plate-counting method; ✓ Bacterialmorphology: SEM;	SP-PDA-Ag/GS-Silk showed reliable antibacterial capacity against *S. aureus* and *E. coli.* It was observed smoothly adhered, proliferated and aggregated bacteria on PEEK, SPEEK and SP-PDA groups;
24. Yuan et al. [59]	Mouse beta-defensin-14 (MBD-14) was immobilized on the PEEK surface with 3D porous structure through sulfonation process;	✓ PEEK—polished, ✓ SP—sulfonated PEEK hydrothermally treated at 120 °C for 4 h (109.11°), ✓ SP-MBD2-SP loaded with 10 uL of solution containing 2 ug/mL MBD-14 (73.40°), ✓ SP-MBD5—SP loaded with 10 uL of solution containing 5 ug/mL MBD-14 (68.25°), ✓ SP-MBD10—SP loaded with 10 uL of solution containing 10 ug/mL MBD-14 (64.80°);	✓ *Staphylococcus aureus* ✓ *Pseudomonas aeruginosa*;	✓ Agar diffusion assay: National Standard of China GB/T 2738-2012 protocol; ✓ Bacterial morphology: SEM; ✓ Antibacterial longevity;	SP-MBD with different MBD-14 solutions could effectively kill *S. aureus* and *P. aeruginosa*;PEEK with MBD-14 exercised durable and broad-spectrum antibacterial activity;
25. Zhang et al. [60]	Macro–microporous bone implants of nano-bioglass (nBG) and polyetheretherketone (PK) composite (mBPC) were fabricated;	✓ Macroporous-microporous nBG/PK composites (mBPC) with the nBG contents of 30 wt %, ✓ PK withoutnBG (mPK), ✓ Macroporous nBG / PK (BPC) compounds with 30% by weight of nBG, ✓ Thiol (HK) loaded in mBPC (dmBPC);	✓ *Staphylococcus aureus*;	✓ The number of CFUs on medium and on biofilm was counted; ✓ Antibacterial activity: LIVE/DEAD Bac light Bacteria Viability Kits (evaluation at CLSM);	Thiol (HK) loaded in mBPC (dmBPC) inhibited *S. aureus* growth and no viable bacteria were found. The presence of higher bacteria number on macro–microporous nBG/PK composites indicated stimulation of bacterial growth/ adhesion;
